# Gene set analysis controlling for length bias in RNA-seq experiments

**DOI:** 10.1186/s13040-017-0125-9

**Published:** 2017-02-06

**Authors:** Xing Ren, Qiang Hu, Song Liu, Jianmin Wang, Jeffrey C. Miecznikowski

**Affiliations:** 1 Department of Biostatistics, SUNY University at Buffalo, Buffalo, 14214 USA; 2 Department of Biostatistics and Bioinformatics, Roswell Park Cancer Institute, Buffalo, 14263 USA

**Keywords:** RNA-seq, Gene set analysis, Gene length bias, Maxmean statistic, Restandardization

## Abstract

**Background:**

In gene set analysis, the researchers are interested in determining the gene sets that are significantly correlated with an outcome, e.g. disease status or treatment. With the rapid development of high throughput sequencing technologies, Ribonucleic acid sequencing (RNA-seq) has become an important alternative to traditional expression arrays in gene expression studies. Challenges exist in adopting the existent algorithms to RNA-seq data given the intrinsic difference of the technologies and data. In RNA-seq experiments, the measure of gene expression is correlated with gene length. This inherent correlation may cause bias in gene set analysis.

**Results:**

We develop SeqGSA, a new method for gene set analysis with length bias adjustment for RNA-seq data. It extends from the R package GSA designed for microarrays. Our method compares the gene set maxmean statistic against permutations, while also taking into account of the statistics of the other gene sets. To adjust for the gene length bias, we implement a flexible weighted sampling scheme in the restandardization step of our algorithm. We show our method improves the power of identifying significant gene sets that are affected by the length bias. We also show that our method maintains the type I error comparing with another representative method for gene set enrichment test.

**Conclusions:**

SeqGSA is a promising tool for testing significant gene pathways with RNA-seq data while adjusting for inherent gene length effect. It enhances the power to detect gene sets affected by the bias and maintains type I error under various situations.

## Background

Ribonucleic acid sequencing (RNA-seq) is a revolutionary tool for gene expression profiling. It has become an important alternative to traditional expression arrays in varieties of studies. How to adopt the existent algorithms for expression arrays to RNA-seq data is a challenge in data analysis. In microarrays the gene expression is a continuous number, while in RNA-seq it is a non-negative integer indicating the number of reads of a gene. More importantly some inherent biases in RNA-seq experiments need to be accounted for. Given the protocol of RNA-seq, it is reasonable to expect that a longer gene will have more counts than an equally expressed short gene. The length effect will cause bias in gene set analysis [1–3].

**Fig. 1:**
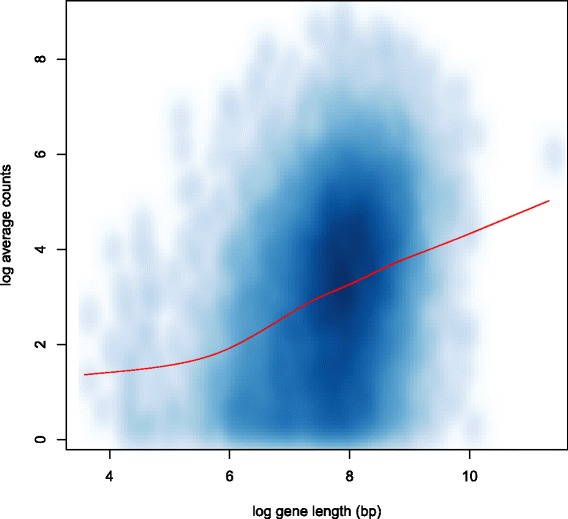
Correlation of count and gene length. The scatterplot of gene length vs average counts (log-log scale) for genes in the LNCaP data set [4]. *Darker color* indicates higher scatter density. The *red curve* is a smooth spline with 7 degrees of freedom, showing a positive correlation between count and gene length

**Fig. 2:**
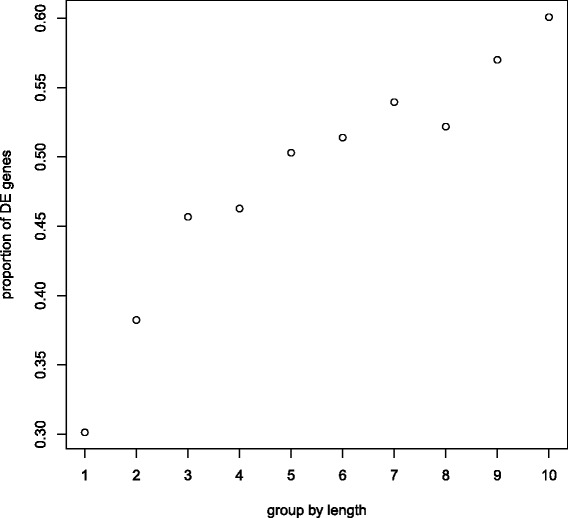
Gene length bias in RNA-seq data. The LNCaP data set [4] shows the probability of significant *p*-values (*p*<0.05) increases as gene length. Group 1 is the genes of shortest length and Group 10 is the genes with longest length

We use the lymph node carcinoma of the prostate (LNCaP) cells RNA-seq data set [4] as an example to show the gene length bias. The data set contains the RNA sequencing profiles of 3 androgen-treated samples and 4 control samples. For every gene we calculate the average count of the 7 samples. Figure 1 shows the smooth scatterplot of gene length vs average count on a log-log scale. A smooth spline is fit to the scatterplot, suggesting a strong positive correlation between counts and gene length. The dependence of counts and gene length introduces a bias in the test of DE genes such that discoveries will favor long genes over short genes. We rank the genes by their length and divide them into 10 groups, with each group containing 10% of the total genes. We use the exact test in the R package edgeR to obtain the *p*-value (unadjusted) for every gene. Figure 2 shows the percentage of DE genes (*p*-value <0.05) in each group increases with gene length. Given the definition in (1), a set consisting of primarily long genes will have a greater maxmean statistic than an equally expressed set of short genes. As a result, the test will have bias that favors sets of long genes.

There are a number of well-established gene set analysis methods for expression arrays. Most of these tests can be roughly classified into two groups, over-representation analysis (ORA) and functional class scoring (FCS) [5]. In ORA, the genes are labeled as differentially expressed (DE) or null based on thresholding their test statistics or *p*-values. Then we test the gene sets for over-representing the DE genes. The most common tests are based on hypergeometric, Chi-square or binomial distribution [5]. Despite the wide usage, ORA approaches have a few limitations. First, some information is discarded in ORA as it ignores the continuous measurement of the test statistics and treats the data as binary outcomes (DE or null). Second, the test for over-representation assumes the genes are independent of each other, which is unlikely to hold given the complex interactions between genes, especially for genes in the same pathway.

The FSC approaches overcome the aforementioned limitations of ORA. First the test statistics are computed for individual genes, e.g. correlation [6], ANOVA [7], Q-statistic [8], signal-to-noise ratio [9], t-statistic [7, 10], z-statistic [11]. Then these gene-level statistics are aggregated into a pathway-level summary statistic e.g. Kolmogorov-Smirnov statistic [9, 12], sum, mean or median [13], the Wilcoxon rank sum [14] and the maxmean statistic [15]. By keeping the gene-level statistics, FSC can detect weak but coordinated changes in gene pathways. In assessing the significance of the pathway statistic, the hypothesis test can be classified into two major categories, self-contained or competitive. A self-contained test permutes sample labels and compares the pathway with its permutations, while a competitive test randomizes genes for each pathway, and compares the pathway with others. The self-contained test maintains the correlation structure between genes, but ignores the other pathways. On the other hand, the competitive test takes into account the other pathways but ignores the gene correlation.

Several gene set/pathway analysis methods have been developed for RNA-seq data, e.g. GOSeq [16], GSVA [17], SeqGSEA [18], CAMERA [19]. GOSeq employs a revised hypergeometric test for DE gene enrichment with the sampling probability adjusted to gene length. GSVA estimates variation of pathway activity over a sample population and it is able to detect subtle changes of gene expression in the pathway. SeqGSEA adopts DSGseq [20] and DESeq [21] for gene level test and uses the GSEA method [9] for functional enrichment analysis. CAMERA [19] estimates the inter-gene correlation and accordingly adjusts the gene set test statistic.

We propose a new functional scoring method SeqGSA for gene set analysis with RNA-seq data. Our method adopts the maxmean statistic in R package GSA [15] as the gene-set-level statistic. Comparing with the more commonly used method GSEA [9], it is argued that the maxmean statistic used in GSA is more powerful than the Kolmogorov-Smirnov statistic in GSEA [15]. The fundamental idea of GSA is that it combines the features of both self-contained test and competitive test through a restandardization procedure. As the original GSA, our method maintains the correlation of genes in permutation tests while also taking into account the competition of other random gene sets. We implement a flexible weighted restandardization scheme to adjust for the gene length bias. It works with any gene-level DE test e.g. [21–23]. We show in the presence of a length bias our method improves the power of identifying significant gene sets and controls type I errors. We also compare our method with GOSeq [16] and CAMERA [19], two representative methods for gene set enrichment test. We show that the maxmean statistic is more appropriate in detecting small changes of expression. Our method also maintains the type I error more accurately when genes are correlated.

## Methods

### Maxmean statistic and restandardization in GSA

In GSA the gene-level test statistics are first converted to *z* statistics using quantile functions, and then the *z* values are aggregated into a gene-set-level maxmean statistic. A restandardization procedure compares the maxmean statistic against permutations while also taking into account sets formed by random selections of genes. Given gene-level *z* statistic *z*_*i*_,*i*=1,…,*n*, let $\mathcal {S}$ be the indices of the gene set and $n_{\mathcal {S}}$ be the size of $\mathcal {S}$. The maxmean statistic *S* is defined as,

1 $$ \begin{aligned} S_{+} & = \frac{1}{n_{\mathcal{S}}} \sum_{i \in \mathcal{S}} z_{i} I\{z_{i} > 0\} \, {,} \\ S_{-} & = -\frac{1}{n_{\mathcal{S}}}\sum_{i \in \mathcal{S}} z_{i} I\{z_{i} < 0\} \, {,} \\ \mathrm{S} & = \max(S_{+}, S_{-}) \, {.} \end{aligned}  $$

Assessing statistical significance of the maxmean statistic requires a null distribution. There are two operations considered to obtain such null distribution, randomization or permutation. For randomization, the null is constructed by randomly sampling a large number of gene sets (row sampling). The problem of randomization is that random sets do not maintain the correlation structure among genes as in real sets. For permutation, the null is estimated by column (sample labels) permutations so that genes still remain in the same set. It maintains the correlation structure. The problem of permutation is that it ignores the distribution of the maxmean statistics of the other gene sets. The restandardization procedure in GSA combines randomization and permutation. Given *z* statistic *z*_*i*_(*i*=1,…,*n*) and gene set $\mathcal {S}$ with $n_{\mathcal {S}}$ genes, the algorithm works as follows,

1. Randomization: calculate the mean and standard deviation of the maxmean statistics of randomized gene sets, denoted by *μ* ^*†*^ and *σ* ^*†*^.
2. Standardization: compute standardized maxmean *S* ^∗^, *S* ^∗^=(*S*−*μ* ^*†*^)/*σ* ^*†*^.
3. Permutation: permute sample labels *B* times and compute the standardized maxmean for each permuted data as in 1) and 2), *S* ^∗*b*^,*b*=1,…,*B*.

To assess how significant a gene set is associated with the outcome, the *p*-value is calculated by comparing *S* ^∗^ against the permutations, $p = \sum _{b=1}^{B} I\{ S^{*b} > S^{*}\}/B$. With restandardization, the null distribution for *S* is essentially the permuted maxmean statistic rescaled by *σ* ^*†*^ and relocated by *μ* ^*†*^. Therefore the maxmean statistic is compared against the permutations while also considering the distribution of the randomized sets.

### Restandardization weighted by gene length

In the randomization step of GSA, all genes are sampled with equal probability. In particular, two sets with the same number of genes will have the same *μ* ^*†*^ and *σ* ^*†*^ regardless of the length of genes in the set. As the method was originally developed for microarrays, it does not consider the potential bias in RNA-seq, as shown in the introduction. To adjust for gene length bias we propose a weighted restandardization algorithm. The weighted algorithm uses the similarity of gene length as sampling weight in the randomization step of the restandardization. The empirical cumulative distribution function (CDF) of the gene length is:

2 $$ \hat{F}(x) = \sum\limits_{i=1}^{n} I\{l_{i} \le x\}/n \, {,}  $$

where *l*_*i*_ is the number of base pairs of gene *i* and *n* is the total number of genes. Note *l*_*i*_ for gene length can be retrieved from public databases e.g. Ensembl (http://www.ensembl.org/) and refSeq (http://www.ncbi.nlm.nih.gov/refseq/).

For a set $\mathcal {S}$, the process of constructing randomized set can be viewed as stepwise replacing genes in $\mathcal {S}$ with genes from all the genes. Instead of assigning equal probability to all genes, we let *q*_*ij*_ be the probability of replacing gene *i* in $\mathcal {S}$ by gene *j*, which is weighted by $1-\vert \hat {F}(l_{i})-\hat {F}(l_{j}) \vert $,

3 $$ \begin{aligned} w_{ij} &= 1-\vert\hat{F}(l_{i})-\hat{F}(l_{j})\vert \quad \forall \quad i \in \mathcal{S} \quad \text{and} \quad j = 1,\ldots,n \, {,} \\ q_{ij} & = \frac{w_{ij}}{\sum_{j=1}^{n} w_{ij}} \, {.} \end{aligned}  $$

The probability *q*_*ij*_ is large when gene *i* and gene *j* have similar length and it is small when their lengths are very different. For a set $\mathcal {S}$, let $q_{\mathcal {S}j}$ be the probability of selecting gene *j* (*j*=1,2,…,*n*) into a randomized set. Since $n_{\mathcal {S}}<<n$ in practice, the following approximation can be made,

4 $$ q_{\mathcal{S} j} \approx \frac{\sum_{i \in \mathcal{S}} q_{ij}}{\sum_{j=1}^{n} \sum_{i \in \mathcal{S}} q_{ij}} \, {.}  $$

Random sets are constructed from a multinomial distribution with probability $q_{\mathcal {S}j}$ for gene *j*. To differentiate from *μ* ^*†*^ and *σ* ^*†*^ in the previous section, we denote the mean and standard deviation of weighted randomization by $\mu _{w}^{\dagger }$ and $\sigma _{w}^{\dagger }$. We use $\mu _{w}^{\dagger }$ and $\sigma _{w}^{\dagger }$ to standardize the maxmean statistic of $\mathcal {S}$,

5 $$ S_{w}^{*} = \left(S - \mu_{w}^{\dagger}\right)/\sigma_{w}^{\dagger} \, {.}  $$

Permutation maxmean statistics $S_{w}^{*b}, b = 1, \ldots, B,$ are computed in the same fashion from permuted data sets. The *p*-value for $\mathcal {S}$ is calculated by comparing $S_{w}^{*}$ and $S_{w}^{*b}$,

6 $$ p = \sum\limits_{b=1}^{B} I\left\{ S_{w}^{*b} > S_{w}^{*}\right\}/B \, {.}  $$

### Justification of weighted restandardization

In this section we present a model for RNA-seq testing that accommodates a gene length bias. Under the gene length bias, we show our weighted method has more power to detect gene sets consisting of short length genes.

We begin with the two group model proposed in [24] where we assume there are *n* cases (genes) that are each either null or non-null with probability *p*_0_ and *p*_1_ and with *z*-values having density either *f*_0_(*z*) or *f*_1_(*z*). We assume *f*_0_(*z*) is the standard normal density and *f*_1_(*z*) is some no-null density. As mentioned in previous section, RNA-seq tests are not based on traditional *z*-values and hence we transform the *p*-values to *z*-values, via *z*_*i*_=*Φ* ^−1^(*p*_*i*_) where *Φ*(·) is the standard normal CDF. We have,

7 $$ z_{i} \sim f(z) = p_{0} f_{0}(z) + p_{1} f_{1}(z) \, {,}  $$

where *f*(*z*) is the mixture density composed of *f*_0_(*z*) and *f*_1_(*z*). The setting in (7) has proven very useful in microarray analysis, e.g. see [25–28]. To obtain more specific results we also assert the assumption of independence that *z*_*i*_’s are independent.

We propose the following twist on this model for RNA-seq analysis. Assume genes are ordered according to the gene length, then in light of RNA-seq, we propose the following more general model,

8 $$ z_{i} \sim p_{0} f_{0}(z) + p_{1} f_{1i}(z) \, {.}  $$

In short, we allow each gene to follow a different alternative distribution. This flexibility allows us to characterize a gene length bias.

To accommodate the gene length into the model in (8), we assume *f*_1*i*_ has the following form,

9 $$ f_{1i}(z) = {rf}_{1i}^{-}(z) + (1-r)f_{1i}^{+}(z) \, {,}  $$

where $f_{1i}^{-}$ and $f_{1i}^{+}$ are densities with means $\mu _{1i}^{-} < 0$ and $\mu _{1i}^{+} > 0$, respectively. To impose the gene length bias we will further require

10 $$ \mu_{1i}^{+} \geq \mu_{1j}^{+} \text{ and } \mu_{1i}^{-} \leq \mu_{1j}^{-} \quad \forall \quad i>j \, {.}  $$

For our purposes it is not important to determine the parameters in (9) and (10), however, we will need the assumption in (10) to justify our method. The specification in (10) allows us to handle a gene length bias where, by nature of a gene’s length, it is more likely to have extreme *z* values than a DE gene of shorter length. For simplicity in the following calculations we also assume *f*_1*i*_ is symmetric, that is,

11 $$ f_{1i}(z) = f_{1i}(-z) \quad \forall \quad z \, {.}  $$

The assumption in (11) is plausible, since a priori, it is reasonable to expect a DE gene to be equally likely to be overexpressed as it is to be underexpressed between two conditions. We proceed in the following manner, we derive the asymptotic distribution of the maxmean statistic in (1). We then examine the restandardization procedure and show that our method yields a more accurate restandardization mean for gene sets.

Under (7), by the Lindeberg-Feller theorem [29] if the Lindeberg condition holds, *S*_+_ and *S*_−_ in (1) asymptotically follow a bivariate normal distribution for adequately large $n_{\mathcal {S}}$,

12 $$ \begin{aligned} \left(\begin{array}{l} S_{+} \\ S_{-} \end{array}\right) \sim N_{2}\left\{ \left(\begin{array}{l} \mu_{+} \\ \mu_{-} \end{array}\right) {,} \left(\begin{array}{cc} \sigma_{+}^{2} & \text{Cov}(S_{+},S_{-}) \\ \text{Cov}(S_{+},S_{-}) & \sigma_{-}^{2} \end{array}\right) \right\} \, {,} \end{aligned}  $$

where *μ*_+_=*E*(*S*_+_) and $\sigma _{+}^{2} = V(S_{+})$ and similarly for *S*_−_. Note that under (8)

13 $$ \begin{aligned} \mu_{+} & = p_{0} \int_{0}^{\infty} z\phi(z)dz + \frac{p_{1}}{n_{\mathcal{S}}} \sum_{i \in \mathcal{S}} \int_{0}^{\infty} {zf}_{1i}(z) dz \\ & = p_{0}\sqrt{\frac{2}{\pi}} + \frac{p_{1}}{n_{\mathcal{S}}} \sum_{i \in \mathcal{S}} \int_{0}^{\infty} {zf}_{1i}(z) dz \, {,} \\ \end{aligned}  $$

and due to the symmetry of *f*_1*i*_, *μ*_−_=*μ*_+_.

For completeness the other quantities in (12) are,

14 $$ \begin{aligned} \sigma_{+}^{2} = \sigma_{-}^{2} & = \left(\frac{1}{n_{\mathcal{S}}}\right)^{2} \sum_{i \in \mathcal{S}} V\left(z_{i}^{+}\right) \\ & = \left(\frac{1}{n_{\mathcal{S}}}\right)^{2} \sum_{i \in \mathcal{S}} \left[E \left(\left(z_{i}^{+}\right)^{2}\right) - \left(E \left(z_{i}^{+}\right)\right)^{2}\right] \\ & = \left(\frac{1}{n_{\mathcal{S}}}\right)^{2} \sum_{i \in \mathcal{S}} \left[\left({\vphantom{\sqrt{\frac{2}{\pi}}}} \int_{0}^{\infty} \frac{\sqrt{z}}{2}\left(p_{0}\phi (\sqrt{z}) + p_{1} f_{1i} (\sqrt{z}) \right)dz \right) \right. \\ & \quad \left. - \left(p_{0} \sqrt{\frac{2}{\pi}} + p_{1} \int_{0}^{\infty} {zf}_{1i}(z)dz \right)^{2}\right] \end{aligned}  $$

15 $$ \begin{aligned} \text{Cov}(S_{+},S_{-}) &= E(S_{+}S_{-}) - E(S_{+})E(S_{-}) \\ &= -\left(\frac{1}{n_{\mathcal{S}}}\right)^{2} E \left[\left(\sum_{i \in \mathcal{S}} z_{i} I\left(z_{i}>0 \right)\right) \left(\sum_{i \in \mathcal{S}} z_{i} I\left(z_{i}<0 \right)\right)\right] - (\mu_{+})^{2}\\ &= -\left(\frac{1}{n_{\mathcal{S}}}\right)^{2} \left[\sum_{i \neq j \in \mathcal{S}} E\left(z_{i} z_{j} I\left(z_{i}>0 \right) I\left(z_{j}<0 \right)\right)\right] -\left(\mu_{+}\right)^{2}\\ &= -\left(\frac{1}{n_{\mathcal{S}}}\right)^{2}\left[\sum_{i \neq j \in \mathcal{S}} E\left(z_{i} I\left(z_{i}>0\right)\right) E\left(z_{j} I\left(z_{j}<0\right)\right)\right] -\left(\mu_{+}\right)^{2}\\ &= -\left(\frac{1}{n_{\mathcal{S}}}\right)^{2} \left[\sum_{i \neq j \in \mathcal{S}} \left(p_{0}\sqrt{2/\pi} +p_{1}\int_{0}^{\infty}{zf}_{1i}(z)dz\right)\right. \\ & \quad \times \left. \left(p_{0}\sqrt{2/\pi} +p_{1}\int_{-\infty}^{0}{zf}_{1j}(z)dz\right)\!{\vphantom{\sum_{i \neq j \in \mathcal{S}}}}\right] - \left(\mu_{+}\right)^{2} \end{aligned}  $$

From the work in [30, 31] on order statistics, the pdf of *S* is derived as,

16 $$ \begin{aligned} f_{S}(s) & = \frac{1}{\sigma_{+}} \phi\left(\frac{-s+\mu_{+}}{\sigma_{+}} \right) \times \Phi\left(\frac{\eta \left(-s+\mu_{+} \right)}{\sigma_{+}\sqrt{1-\eta^{2}}} - \frac{-s+\mu_{-}}{\sigma^{-}\sqrt{1-\eta^{2}}}\right) \\ & \quad + \frac{1}{\sigma_{-}} \phi\left(\frac{-s+\mu_{-}}{\sigma_{-}}\right) \times \Phi\left(\frac{\eta(-s+\mu_{-})}{\sigma_{-}\sqrt{1-\eta^{2}}} - \frac{-s+\mu_{+}}{\sigma_{+}\sqrt{1-\eta^{2}}}\right) \end{aligned}  $$

where *η* is the correlation of *S*_+_ and *S*_−_, *ϕ* is the pdf for the standard normal distribution. The mean of *S* (computed via moment generating functions as in [32]) is

17 $$ E(S) = \mu_{+} \Phi\left(\frac{\mu_{+}-\mu_{-}}{\theta}\right) + \mu_{-} \Phi\left(\frac{\mu_{-}-\mu_{+}}{\theta}\right) + \theta \phi\left(\frac{\mu_{-}-\mu_{+}}{\theta}\right),  $$

where $\theta = \left (\sigma _{+}^{2} - 2\eta +\sigma _{-}^{2}\right)^{1/2}$ and $\sigma _{+}^{2}$, $\sigma _{-}^{2}$ and *η* are given in (14) and (15). Hence, we can simplify (17) such that

18 $$ \begin{aligned} E(S) = & \frac{1}{2} (\mu_{+}) + \frac{1}{2} (\mu_{-}) + \theta \phi(0),\\ = & \mu_{+} + 0.40 \theta \\ = & p_{0}\sqrt{\frac{2}{\pi}} + \frac{p_{1}}{n_{\mathcal{S}}} \sum_{i \in \mathcal{S}} \int_{0}^{\infty} {zf}_{1i}(z) dz +0.40\theta \, {,} \end{aligned}  $$

where each quantity in (18) has been previously derived.

We would like *S* to be large for DE sets and small for null sets. Given (18) we see that, on average, *S* will be large for a set of long genes in the presence of a strong gene length bias where $\int _{0}^{\infty } {zf}_{1i}(z)dz$ increases with the length of gene *i*. This event does not represent a true biological gene set enrichment and thus we would consider detecting this set a false positive. On the other hand, *S* could also be large if *p*_1_ is large for the genes in $\mathcal {S}$ relative to *p*_1_ for the genes not in $\mathcal {S}$. This situation does reflect true biological enrichment and therefore we would like our procedure to detect significance in this setting.

So in light of the magnitude of *S* and its randomized distribution for comparison here is the problem: consider a **biologically enriched** gene set $\mathcal {S}$ consisting of primarily short genes in the presence of a strong gene length bias. While *p*_1_ is large for $\mathcal {S}$ it will not be detected because $1/n_{\mathcal {S}} \sum _{i \in \mathcal {S}} \int _{0}^{\infty } {zf}_{1i}(z) dz$ is relatively large for randomly assembled gene sets $\mathcal {S}^{\prime }$. Likewise, for null sets with long genes $\int _{0}^{\infty } {zf}_{1i}(z)dz$ will be large relative to other random sets and we would like a scheme with a low probability of calling this null set significant. Our weighted randomization scheme is designed to increase (relative to the unweighted procedure) the probability of detecting the truly enriched gene set while decreasing the probability of detecting the unenriched gene sets.

Recall the algorithm compares the maxmean statistic against a distribution of permuted maxmean statistics that are centered and scaled according to the mean (*μ* ^*†*^) and variance (*σ* ^*†*^) of maxmean statistics chosen at random. Hence in order for $\mathcal {S}$ to be enriched, *S* must be significantly different than permuted maxmean values *and* random maxmean values. Hence, in light of the scheme above, we would like *μ* ^*†*^ to be small for truly differentially enriched sets and large for truly null sets. Heuristically the main difference between the two methods is in the computation of $\mu _{w}^{\dagger }$ and *μ* ^*†*^. Under the weighted resampling scheme, with *K* resamplings, we have

19 $$ \mu_{w}^{\dagger} = \frac{1}{K} \sum\limits_{k = 1}^{K} S_{w}^{\prime k} \, {.}  $$

Hence, the difference between $\mu _{w}^{\dagger }$ and *μ* ^*†*^ can be studied by examining $E(S_{w}^{\prime k})$ against *E*(*S* ^′*k*^) from the unweighted approach.

From (18) we have

20 $$ E(S_{w}) = \mu_{+w} + 0.40 \theta_{w}  $$

where for the unweighted resampling we have,

21 $$ E(S) = \mu_{+} + 0.40 \theta  $$

We expect the difference between the second order effects *θ*_*w*_ and *θ* will be minor relative to the first order effects (*μ*_+*w*_, *μ*_+_) between the two methods.

From (13) with the weighted scenario we have

22 $$ \mu_{+w} = p_{0} \sqrt{\frac{2}{\pi}} + \frac{p_{1}}{n_{\mathcal{S}^{\prime}_{w}}} \sum_{i \in \mathcal{S}^{\prime}_{w}} \int_{0}^{\infty} {zf}_{1i}(z) dz{,}  $$

where the weighted scenario is based on choosing genes in $\mathcal {S}^{\prime }_{w}$. The unweighted scenario is equivalent except the genes are from $\mathcal {S}^{\prime }$. By design, the genes in $\mathcal {S}^{\prime }_{w}$ are chosen to have a similar length to the genes in $\mathcal {S}$, while the genes in $\mathcal {S}^{\prime }$ are chosen with equal probability. Hence if *i*<*j* then in the presence of a gene length bias in (9) and (10), we have $\int _{0}^{\infty } {zf}_{1i}(z) dz < \int _{0}^{\infty } {zf}_{1j}(z) dz$. Therefore, if the weighted process is biased to choose genes of smaller length relative to the unweighted process, we are likely to obtain *μ*_+*w*_<*μ*_+_. Hence, on average $S^{\prime }_{w}$ will be smaller relative to the unweighted scenario of *S* ^′^ and thus the *p*-values in the weighted approach will be larger.

This feature produces the following effect: for enriched gene sets consisting primarily of short length genes, the weighted restandardization procedure will be more powerful than the unweighted procedure, while in the same way, the weighted method may also increase the type I error. On the surface this does not seem like an appealing solution. However, we will show in several simulations, our method increase the power while still maintaining the type I error under the controlled level.

## Results and discussion

### Simulation

To assess our method, we compare the type I error and power of the two versions of SeqGSA (weighted restandardization and the unweighted restandardization). The unweighted version is similar to the original GSA, except that the *t* test is replaced with the exact negative binomial test of edgeR, as the latter is considered a more appropriate test for RNA-seq count data. In addition, we compare SeqGSA with two gene set test methods, GOSeq [16] and CAMERA [19]. GOSeq tests for higher proportion of DE genes in a set using a modified hypergeometric test, with adjustment to gene length bias. CAMERA compares the *t* statistics of genes inside the set and genes outside the set with adjustment to the intra-correlation of the gene sets. It is embedded in the widely used limma package [33]. We conduct two simulations. In simulation 1, the count data is generated from a Poisson model in which the mean parameter is associated with gene length. The gene sets are constructed without consideration to intra-correlation. In simulation 2, we use the data of an RNA-seq experiment. The gene sets are constructed such that genes in a set are correlated. In both simulations, we let the size of gene set $n_{\mathcal {S}} = 50$. For GOSeq, the enrichment test requires a threshold of the exact test *p*-values for DE genes. For our simulations, the threshold is determined by controlling false discovery rate at 0.1 with the Benjamini-Hochberg procedure [34].

#### Simulation 1: sets of uncorrelated genes

We randomly sample *n*=1000 genes from the LNCaP data set [4] with gene length between 1000 and 3000 base pairs. Let *l*_*i*_(*i*=1,2,…,1000) be the length of gene *i*. The 1000 genes are grouped into 20 non-overlapping sets according to gene length, with 50 genes in every set. Thus set 1 contains the shortest 50 genes, set 2 contains the next shortest 50, and so on. This would generate length bias in gene set analysis. For each set, we let some number of genes be up-regulated and down-regulated. Let *μ*_*i*_=*l*_*i*_/10. In control samples, count *x*_*ij*_∼*P* *o* *i* *s* *s* *o* *n*(*μ*_*i*_). In treatment samples, *x*_*ij*_∼*P* *o* *i* *s* *s* *o* *n*((1+*β*)*μ*_*i*_) for up-regulated genes, *x*_*ij*_∼*P* *o* *i* *s* *s* *o* *n*((1−*β*)*μ*_*i*_) for down-regulated genes and *x*_*ij*_∼*P* *o* *i* *s* *s* *o* *n*(*μ*_*i*_) for null genes, where *β*(*β*=0.15,0.3) indicates the effect of DE at the gene level.

We first compare the type I error. Let *n*_*de*_=2 be the number of up- and down-regulated genes for all 20 sets. Thus all sets are considered as null. Table 1 summarizes the type I error (*p*-value <0.05) in selected sets. Results shows the type I error is under control for all sets. In particular, under small DE effect (*β*=0.15), comparing with the unweighted method, weighted SeqGSA reduces the type I error in sets of long genes (set 15 and set 20), but increases the error in sets of short genes (set 1). GOSeq has higher type I error for set 1 but it is very conservative for all other sets. Under large DE effect (*β*=0.3), all methods are conservative in terms of type I error control and the difference is small. Similarly, CAMERA is very conservative under all settings.

**Table 1:** Type I error of simulation 1

	*β*=0.15				*β*=0.3					
	Set 1	Set 5	Set 10	Set 15	Set 20	Set 1	Set 5	Set 10	Set 15	Set 20
Weighted	0.007	0.007	0.006	0.009	0.017	0	0	0	0.001	0.001
Unweighted	0.002	0.007	0.008	0.014	0.022	0	0	0	0	0.003
GOSeq	0.019	0	0	0	0	0	0	0	0	0
CAMERA	0	0	0	0	0	0	0	0	0	0

Type I error of selected sets (*p*-value <0.05) under different *β*. All four methods maintain the type I error under the controlled level (0.05)

Next we compare the power of identifying DE sets that are undermined by the length bias. We let *n*_*de*_ be a greater number (*n*_*de*_=6,8,10) in set 1, thus set 1 becomes the DE set. An ideal test should have small *p*-values for set 1 and non-significant *p*-values for the others. Table 2 shows that the weighted algorithm increases the power of detecting set 1. In particular, SeqGSA (both weighted and unweighted algorithms) is more powerful under small DE effect (*β*=0.15), while GOSeq is more powerful under more significant change of gene expression (*β*=0.3). On the other hand, CAMERA was unable to detect such DE effect, indicating the test statistic of CAMERA is insensitive to small changes of expression under independence.

**Table 2:** Power of simulation 1

	With length bias						No length bias		
	*β*=0.15			*β*=0.3			*β*=0.15		
*n*_*de*_=	6	8	10	6	8	10	6	8	10
Weighted	0.36	0.48	0.77	0.81	0.95	0.98	0.61	0.93	0.96
Unweighted	0.29	0.45	0.75	0.77	0.90	0.96	0.61	0.94	0.97
GOSeq	0.23	0.38	0.56	0.97	1.0	1.0	0.96	1.0	1.0
CAMERA	0.01	0	0	0	0	0	0.02	0.01	0.02

Power of identifying set 1 as DE (*p*-value <0.05) under different *β* and length bias. The weighted SeqGSA increases the power of the unweighted procedure for detecting set 1. SeqGSA is more powerful with small DE effect (*β*=0.15), while GOSeq is more powerful with large DE effect (*β*=0.3). The two procedures of SeqGSA perform similarly under no length bias

In addition we compare the power under no length bias, i.e. genes are grouped randomly thus no length bias exists among the gene sets. In such case, the weighted algorithm does not have advantage over the unweighted version. There is a slight compromise in the power, but the difference is negligible.

#### Simulation 2: sets of correlated genes

In simulation 2, we use a public data set with simulated gene sets that contain length bias. The data is a subset of the human prostate cancer data [35] downloaded from the European Bioinformatics Institute (EBI) server (http://www.ebi.ac.uk). There are 11 normal and 12 tumor samples in the subset data. The reads were aligned to the human genome reference sequence (version b37 from http://www.1000genomes.org) by TopHat v2.0.8 [36] and Bowtie v2.1.0 [37]. Gene length is retrieved with the getlength function in GOSeq.

Genes with average count less than 1 or with unretrievable length are filtered out. The 23396 remaining genes are divided into four groups by length with each group containing 25% genes. We use edgeR to calculate the *z* values and label gene *i* as DE for |*z*_*i*_|>*Φ* ^−1^(0.975), where *Φ* ^−1^ is the quantile function of standard normal distribution. The standard deviations of the *z* values from group 1 to group 4 are 1.76, 2.02, 2.09, 2.08 respectively, showing the scale of *z* values of group 1 is significantly smaller than others. As a result, the proportion of DE genes (|*z*|>*Φ* ^−1^(0.975)) of the group 1 is lower than the others (26.7% vs 31.1%, 33.6%, 32.9%). Gene sets from group 1 will be affected by the length bias.

Different from simulation 1, the sets in simulation 2 are generated with intra-correlation. We first compare the type I error under correlation. A null gene set is constructed as follows. First a null gene is sampled from the null genes in group 1 as the hub gene [38]. The simulated gene set contains $n_{\mathcal {S}}$ genes that are sampled from group 1 that have high correlation with the null hub gene (correlation coefficient >0.4). The correlation violates the independence assumption of GOSeq, resulting in an inflated type I error (0.105). On the other hand, the gene correlation is taken into account in the permutation tests of SeqGSA, thus the type I errors are under control for both the weighted algorithm (0.014) and the unweighted algorithm (0.011).

Due to inflated type I error of GOSeq for correlated gene sets, we only compare SeqGSA to CAMERA. A DE hub gene is sampled from DE genes in group 1. The simulated gene set contains a high proportion of DE genes (40 to 70%) that are sampled from DE genes in group 1 and have high correlation with the DE hub gene (correlation coefficient >0.4). The other genes in the set are sampled from the null genes in group 1. The weighted method improves the power for identifying the simulated DE sets (Table 3). Both the SeqGSA methods have higher power than CAMERA, suggesting the maxmean statistic is more sensitive to subtle change of expression on the gene-set level.

**Table 3:** Power of simulation 2

Proportion of DE genes	0.4	0.5	0.6	0.7
Weighted	0.22	0.53	0.71	0.87
Unweighted	0.16	0.41	0.67	0.73
CAMERA	0.09	0.19	0.26	0.39

Power of identifying DE sets (with correlation) in group 1 (*p*-value <0.05). The DE sets are constructed with a high proportion of DE genes in group 1

#### Simulation 3: power on sets of long genes

As the weighted method samples more often from genes with similar length, we expect it should also result in a decrease of the power on sets with long genes. In simulation 3 we evaluate how significant the effect is. The same strategy of simulation 2 on was run on group 3 and 4 for the long genes. Results show although there is a slight decrease in the power for group 3 and 4 overall, the decrease is very minimal compared with the increase on the short gene sets in simulation 2 (Table 4). Therefore the weighted method should increase the overall power of all gene sets.

**Table 4:** Power of simulation 3

Proportion of DE genes	0.4	0.5	0.6	0.7
Weighted	0.45	0.75	0.90	0.95
Unweighted	0.49	0.76	0.88	0.95

Power of identifying DE sets (with correlation) in group 3 and 4 (*p*-value <0.05)

### Application to RNA-seq data set

**Fig. 3:**
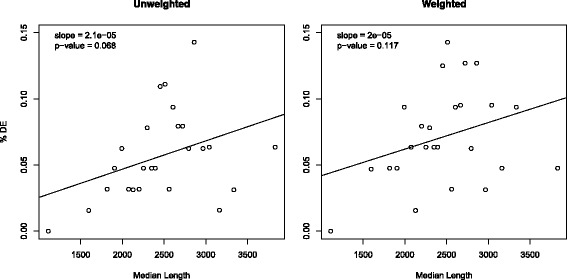
Proportion of DE of GO categories for unweighted and weighted SeqGSA. Genes in the LNCaP data set in [4] are mapped to the GO categories. The 1585 mapped GO categories are grouped by median gene length from low to high. For the unweighted method (*left*), the correlation of the proportion of DE categories and gene length is on the borderline of significance (*p*=0.068). For the weighted method (*right*), the correlation is not significant (*p*=0.117)

In this section we first assess the effectiveness of gene length adjustment of SeqGSA with true biological gene sets. Again we use the LNCaP data set [4]. The genes are mapped to the GO categories [39] via R package biomaRt [40] on ENSEMBL (www.ensembl.org) homo sapiens database. The mapped GO categories are filtered by their size. The 1585 categories with 20 to 200 mapped genes are used for the analysis. Small categories are filtered out to prevent outliers in gene length for small gene sets. Large categories are filtered out to avoid slow computation. These categories are grouped into 25 bins of approximately equal size by their median gene length. Figure 3 compares the correlation between the proportion of DE categories and the median length of the bin. A straight line is fit with the slope and its *p*-value shown on the figure. For the unweighted method the correlation is on the borderline of significance (*p*=0.068) while for weighted method the correlation is not significant (*p*=0.117).

**Fig. 4:**
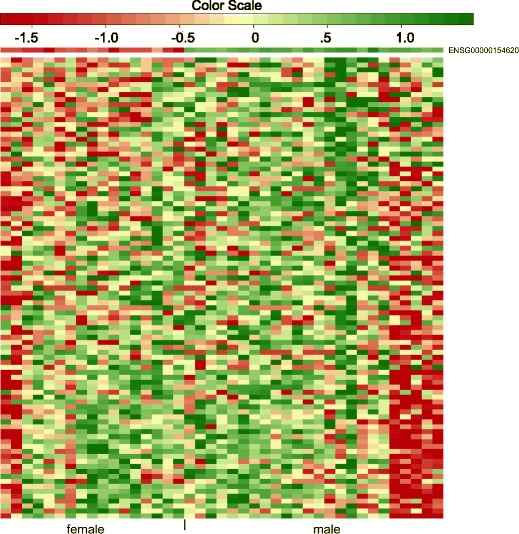
Heatmap of hsa04810 KEGG pathway. The heatmap of the 94 mapped genes in the hsa04810 KEGG pathway for the CEPH HapMap data [41]. The count data is log-transformed and row-scaled

**Fig. 5:**
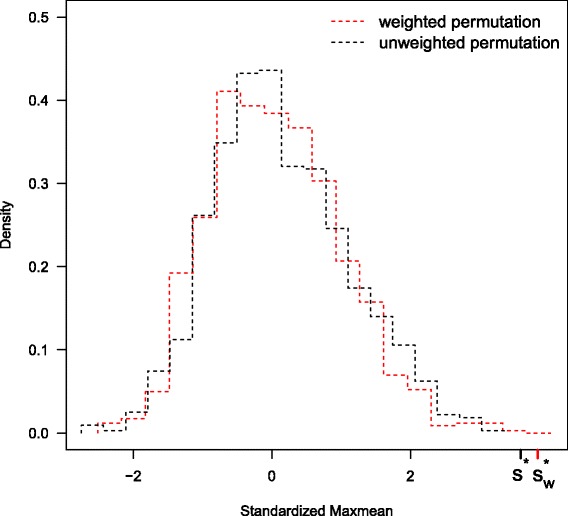
Standardized maxmean and permutations of hsa04810 pathway. The standardized maxmean statistic and permutations of the hsa04810 pathway for the CEPH HapMap data [41]. The standardardized maxmean $S_{w}^{*}$ (*red*) by the weighted method falls on the right of the unweighted *S* ^∗^ (*black*), while the permutation statistics of the two methods overlap

Next we apply the methods to another public RNA-seq data set. The data set is from the Center for the Study of Human Polymorphisms (CEPH) HapMap samples [41]. It contains 17 female and 24 male human B-cell samples. Genes with average count less than 1 or with unretrievable gene length by the getlength function in GOSeq are filtered out. We map the genes to the 229 KEGG pathways via the getgo function in GOSeq. Only 2549 genes can be mapped to the KEGG pathways. We further filter out pathways with 5 or fewer mapped genes. We run SeqGSA and GOSeq to test DE between male and female group on the remaining 208 pathways. Controlling FDR at 0.1, the weighted SeqGSA identifies the hsa04810 pathway, regulation of actin cytoskeleton. The pathway contains 214 genes, 94 of which are mapped to the data set. It is associated with multiple genetic diseases including sex-linked disorders as the non-syndromic X-linked mental retardation. Studies have shown the pathway is related to sex steroids regulation of cell morphology and tissue organization that may play an important role in gender-specific differences of brain dysfunction [42, 43]. Figure 4 is the heatmap of the hsa04810 pathway for the 41 samples. The genes are ordered by their *z*-values. The significance of the pathway is driven by gene TMSB4Y (Ensemble gene ID ENSG00000154620) that lies on the forward strand of chromosome Y. Its homolog on chromosome X escapes X inactivation and encodes an actin sequestering protein (provided by RefSeq, Jul 2008). The TMSB4Y gene is shown in relation to multiple biological activities including actin polymerization and depolymerization in non-muscle cells [44], activation of natural killer cell cytotoxicity [45] and minor histocompatibility antigen encoding [46], etc. The unweighted procedure also identifies the same pathway. Figure 5 compares the standardized maxmean statistic for hsa04810 pathway and its permutation statistics of the two algorithms. The two versions of permutation statistics overlap, while the standardized maxmean $S_{w}^{*}$ (red tick) by the weighted method falls further away from the permutation comparing with the unweighted *S* ^∗^ (black tick), suggesting that our weighted method is more powerful in finding the pathway. On the other hand, GOSeq and CAMERA do not find any pathway at FDR =0.1. This example demonstrates our method is easy to implement and gives favorable results in detection of small but coordinated change in gene sets.

### Discussion

We propose SeqGSA, an extension of the GSA method for RNA-seq data. To calculate maxmean statistics from the RNA-seq count data, we replace the *t* test in the original GSA with a count based test, e.g. the negative binomial test in edgeR. The users can also substitute it with custom defined methods for the gene-level test. More importantly we propose a weighted restandardization approach to accommodate the gene length bias in RNA-seq data. Different from traditional expression arrays, gene length effect generally exists in RNA-seq data sets. To compute the null distribution of the maxmean statistic, GSA employs a randomization strategy that randomly samples genes with equal weights. However, having equal sampling weights ignores the length effect in the data. To adjust for the bias, we use the similarity of gene length as the sampling weight instead, so that the randomized sets will be more likely to consist of genes with similar length. This reduces the bias and yields more accurate null distribution of the maxmean statistics. A DE set comprised mainly of short genes will be more likely called significant in our weighted approach. On the other hand, a null set comprised of long genes will be less significant so that type I error is reduced for these sets. We show by simulations that our method reduces the length bias in RNA-seq data. In addition, we show that the weighted and non-weighted approaches have very similar results when there is no length bias present in the data.

There have been several other methods proposed to remove the length bias in enrichment testing with RNA-seq data [2, 3, 16]. In those works, the enrichment test employs the modified hypergeometric test, Wilcoxon rank sum test or logistic regression. These methods fall in the category of over-representation analysis (ORA) that compares the set against randomized sets. A fundamental difference of our method is that it works under the permutation scheme while also taking into account gene set randomization. An advantage of the permutation test is that it considers the correlation in a gene set and the correlation structure is maintained during sample label permutations. We compare our method against GOSeq in two simulations. GOSeq also implements a procedure for length bias adjustment. In GOSeq, the adjustment occurs on the gene level. A spline is fit to the proportion of DE genes and gene length. This spline is used to correct the sampling probability of the hypergeometric test. In our method, we adjust the bias on the gene set level. We recalculate the randomization mean and standard deviation based on weighted sampling so that it compares against gene sets of similar length. In the simulations, we control the level of DE by the parameter *β*. The value of *β* is chosen to represent two settings, *β*=0.15 for weak DE and *β*=0.30 for moderate DE, each representing the strength of the two methods. In simulation 1, the gene sets are sampled independently. We show that our method is more powerful in finding sets of genes with weak changes. In particular, when expression of genes is weakly changed, the test statistics for many DE genes fall below the threshold of multiple test adjustment in GOSeq. As a result, the power of detecting set of weakly DE genes is undermined. On the other hand, our method aggregates the weak signals of individual genes and increases the power of detecting such DE sets. When there is no correlation among the simulated gene sets, the type I errors of all methods are under the controlled level. In simulation 2, we use the data of an RNA-seq experiment and compare the three methods on simulated gene sets. The gene sets are constructed such that the genes are strongly correlated. This simulates the fact that many of the pre-defined gene sets are identified by gene-gene correlation. The hypergeometric test of GOSeq is based on the assumption that genes are independent. As a result of violated assumption, the type I error of GOSeq is inflated. On the other hand, the permutation test in SeqGSA takes into account the correlation structure within a gene set and thus maintains the type I error more accurately. We also compare SeqGSA with CAMERA, a competitive gene set test also based on sample permutations with adjustment to gene-set intra-correlation. We show that the maxmean statistic in SeqGSA is more sensitive to subtle but synchronized changes in the gene sets, which has been shown as one of the advantages of the original GSA method [15]. In both simulations, CAMERA has limited power to detect small to moderate changes on the gene-set level.

There are some limitations to our length-weighted method. First there are other sources of bias existent in the RNA-seq data, such as “GC-content”. It has been shown that genes with a large number of guanine (G) and cytosine (C) bases are preferentially read by sequencing machines and the effect may not be monotone [47]. A solution to such bias issues is to correct the bias at the gene level by modeling the number of reads or test statistics by gene length or GC content. However this method has its own problem since the true expression level is unknown. Without a comparison experiment, it is difficult to tell whether the difference in reads and test statistics results from true biological expression or biases. Second, the resampling step in SeqGSA weighted by the empirical CDF of gene length is a simple solution, but it does not completely remove the length bias. To improve the performance of the weighted restandardization, we may consider using tunable parameters to adjust the weight. Determining tuning parameters requires further exploration and assumptions. Third, our method improves the power for sets comprised of short genes, but it may slightly compromise the power to detect enriched sets comprised of long genes. This compromise is common for all methods of length bias adjustment. We feel this comprise is scientifically justifiable since the gain in power for small length gene pathways is much larger than the loss of power for large length gene pathways. The effect of this bias on the family-wise error rate or false discovery rate in multiple gene set testing needs further investigation and is part of our future work on this topic. Fourth, in this paper we focus on the exact binomial test in edgeR for gene-level test. However, there are many other tests that can be considered, e.g. see [21, 23, 48–51]. Alternatively, log fold change can be considered as it is not affected by length. This motivates a question for future work on an optimal test statistic for gene set analysis. Last, the computation performance of the weighted algorithm is slightly slower than the unweighted algorithm as it estimate mean and standard deviation using different weights, but the speeds are comparable as the most computationally intense step, computing the permutation *z* values, is the same for both methods.

## Conclusions

We develop a gene set analysis method for RNA-seq data affected by gene length bias. This novel approach is designed to enhance the power to detect DE gene sets comprised of mainly small length genes. Importantly, we justify our method and demonstrate that it controls the type I error comparing to a representative ORA method for RNA-seq. Also, we show that without the presence of a gene length bias, our approach still performs nearly the same as the original unweighted algorithm in GSA. We expect our gene set analysis method will be of great utility to researchers performing gene set analysis with RNA-seq datasets.
